# Supplementary material for: SMARCA4 Depletion Induces Cisplatin Resistance by Activating YAP1-Mediated Epithelial-to-Mesenchymal Transition in Triple-Negative Breast Cancer
Source: Cancers (Basel). 2021 Oct 30;13(21):5474. doi: 10.3390/cancers13215474 (PMC8582548; doi:10.3390/cancers13215474)
Supplement: Supplementary file 1 [file cancers-13-05474-s001.zip › cancers-1372444-supplementary/supplementary_file/cancers-1372444 supplementary material figures.pdf]

cancers

MDPI

Supplementary Material: *SMARCA4* Depletion Induces Cisplatin Resistance by Activating YAP1-Mediated Epithelial-to-Mesenchymal Transition in Triple-Negative Breast Cancer

Jihyun Kim, Gyubeom Jang, Sung Hoon Sim, In Hae Park, Kyungtae Kim * and Charny Park *

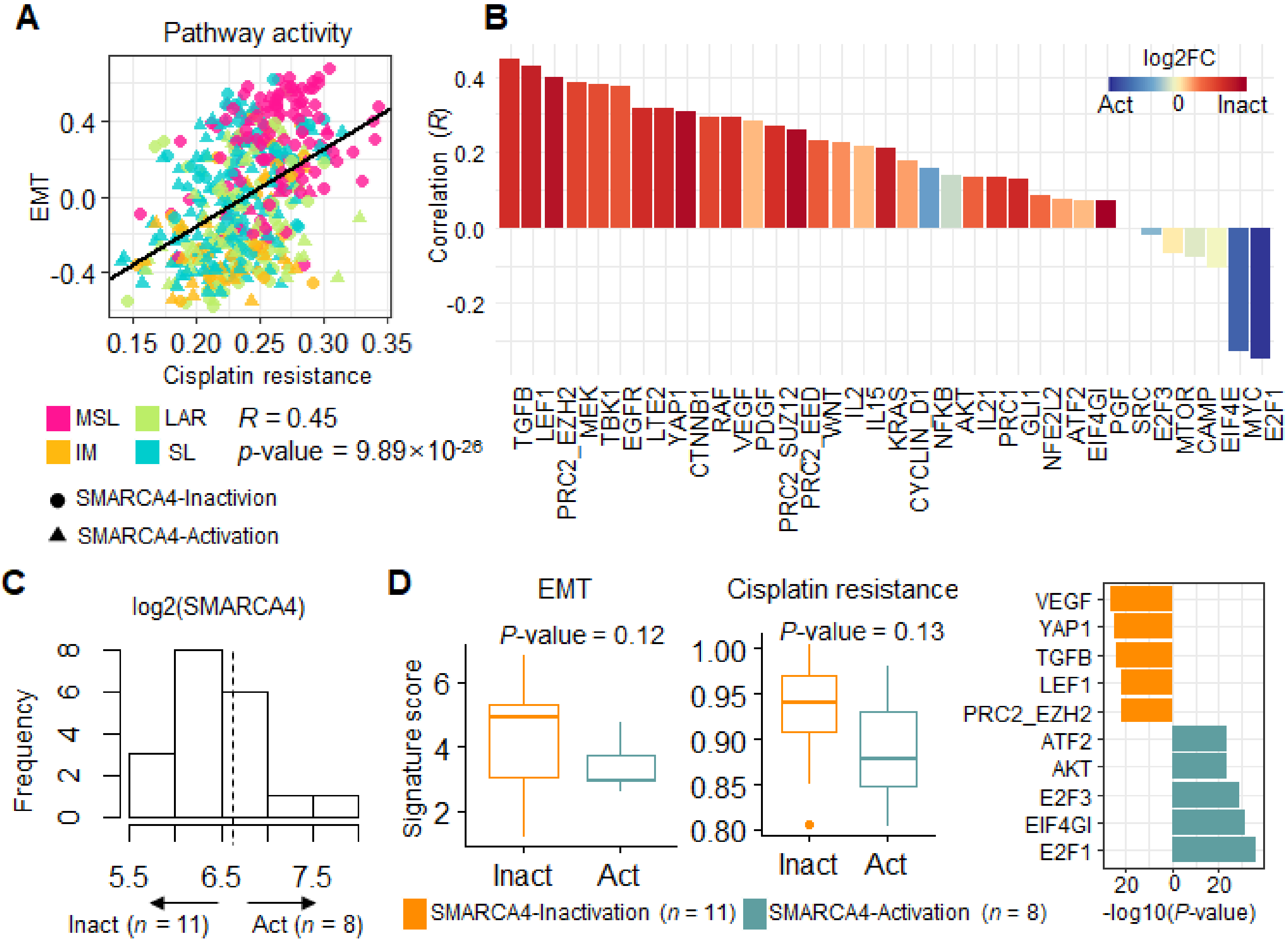

Figure S1. Additional analyses according to SMARCA4 activation. (**A**) Scatter plot of EMT and cisplatin resistance scores. The scores were positively correlated (R = 0.45), and samples of the MSL subtype showed high EMT and cisplatin resistance scores. Circle, SMARCA4 inactivation; triangle, SMARCA4 activation. (**B**) A waterfall plot of correlations between the oncogene signature and EMT scores. Each bar is colored based on the log2FC in expression of the indicated gene between the SMARCA4 activation and inactivation groups. (**C**) Distribution of SMARCA4 expression from CCLE TNBC cell lines. Nineteen TNBC cell lines divided into SMARCA4 activation (n = 8) and inactivation groups (n = 11) based on median value (black dash line). (**D**) Boxplots of gene signature scores in the SMARCA4 inactivation (orange) and activation (teal) groups of TNBC cell lines for EMT and cisplatin resistance signatures. *P*-values were calculated by Wilcoxon rank

sum tests. (**E**) Bar plot of log-scale *P*-values by GSVA limma test with oncogene signature scores according to SMARCA4 inactivation (orange) and activation (teal) groups from TNBC cell lines.

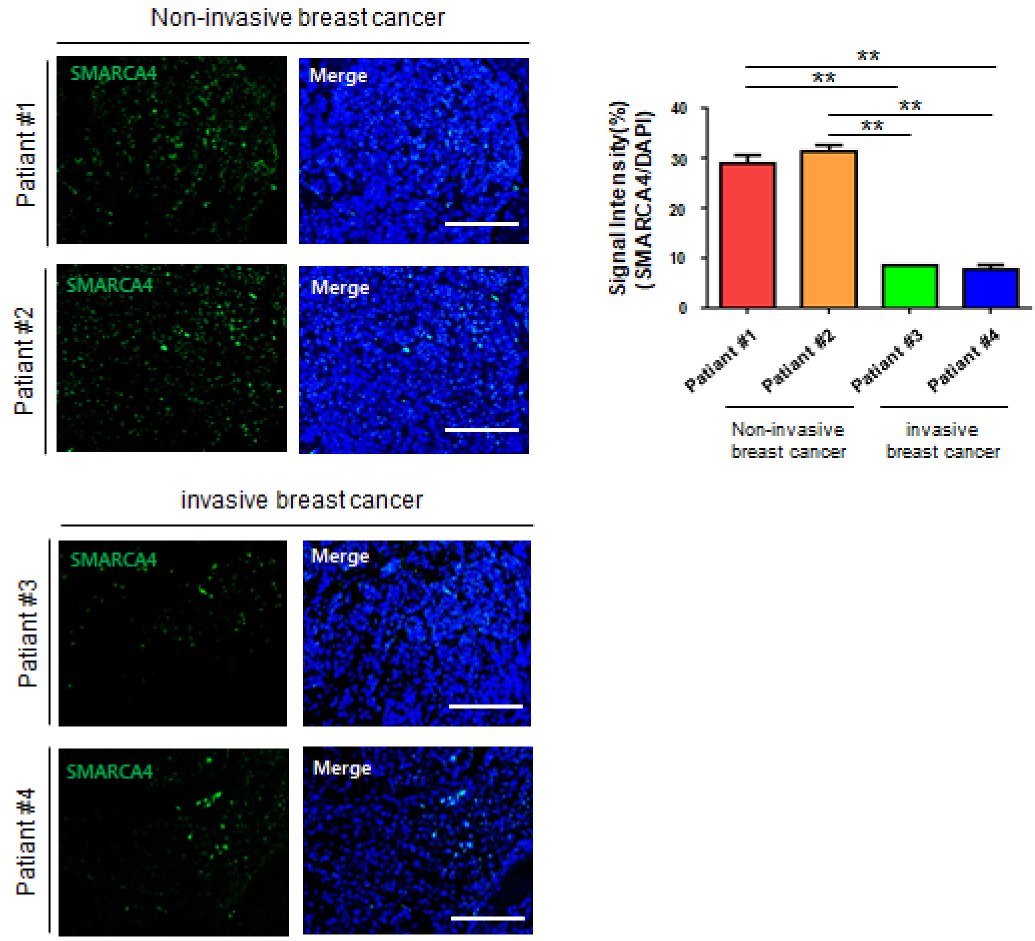

Figure S2. Immunofluorescence analysis of SMARCA4 expression in non-invasive and invasive breast cancer tissues. Non-invasive and invasive breast cancer tissues were stained with antibodies against SMARCA4 (scale bar, 200 μm). The nuclei were visualized using DAPI and representative images are shown on the left. The bar plot shown the signal intensity in SMARCA4 expression on the right. Statistical analyses were carried out using Graph Pad Prism v7.0, and data was analyzed by unpaired t-test ** $p < 0.01$, N.S. = not significant.

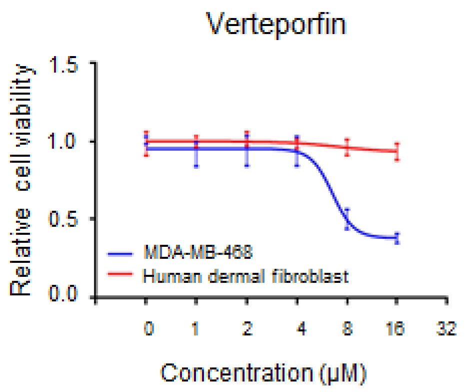

Figure S3. The comparison of cell viability on verteporfin between human dermal fibroblast and TNBC cells. The inhibition of cell viability through treatment with verteporfin for 48h was determined using a CCK-8 assay in MDA-MD-468 cells and human dermal fibroblasts. The cell viability was calculated as fold change of the vehicle control (DMSO).
