# Supplementary material for: SMARCA4 Depletion Induces Cisplatin Resistance by Activating YAP1-Mediated Epithelial-to-Mesenchymal Transition in Triple-Negative Breast Cancer
Source: Cancers (Basel). 2021 Oct 30;13(21):5474. doi: 10.3390/cancers13215474 (PMC8582548; doi:10.3390/cancers13215474)
Supplement: Supplementary file 1 [file cancers-13-05474-s001.zip › cancers-1372444-supplementary/supplementary_file/Supplementary_TableS2.pdf]

Table S2. Primer sequences for quantitative RT-PCR.

Gene	Genebank No.		Primer sequence
**SMARCA4**	NM_001128849	**F**	TTGCCATGGTCCCTCTCGC
		**R**	TGACCGCATCCCCATTCCTT
**Twist**	NM_000474	**F**	CAGCGCACCCAGTCGCTGAA
		**R**	CGCCCCACGCCCTGTTTCTT
**Snail**	NM_005985	**F**	GAGGCGGTGGCAGACTAG
		**R**	GACACATCGGTCAGACCAG
**Slug**	NM_003068	**F**	TGCGATGCCCAGTCTAGAAA
		**R**	GTGTCCTTGAAGCAACCAGG
**N-cadherin**	NM_001792	**F**	CTCCTATGAGTGGAACAGGAACG
		**R**	TTGGATCAATGTCATAATCAAGTGCTGTA
**Claudin-1**	NM_021101	**F**	GAAGTGCTTGGAAGACGATG
		**R**	GAGCCTGACCAAATTCGTAC
**IGFBP3**	NM_001013398	**F**	CCCTGCCGTAGAGAAATGGAA
		**R**	GCCCATACTTATCCACACACCA
**NT5E**	NM_002526	**F**	GGAGAACCTGGCTGCTGTATT
		**R**	ACATGGATTCCGCCCACCT
**GADD45A**	NM_001924	**F**	CTCTTGGAGACCGACGCTG
		**R**	TCCATGTAGCGACTTTCCCG

**TGFB1**	NM_000660	**F**	TGCAGGTATTGATGGCACCT
		**R**	CCGGTTGGTCTGTTGTGACT
**CRIM1**	NM_016441	**F**	TTGTGGAAGGAGAAACGTGGA
		**R**	AAGGAAGGCCGAAAAGGTTGA
**TGFB2**	NM_003238	**F**	TGCAGGTATTGATGGCACCT
		**R**	CCGGTTGGTCTGTTGTGACT
**AMOTL2**	NM_016201	**F**	AGAGCTACGAACAGCAGCAG
		**R**	CCGCTCCACTTTCTCCACAT
**NUAK2**	NM_030952	**F**	GTGACACGGCTGATGACACT
		**R**	AGAATGCCCTTCTTGGGGAG
**CTGF**	NM_001901	**F**	TTACCAATGACAACGCCTCCT
		**R**	AGCTCGGTATGTCTTCATGCT
**GAPDH**	NM_002046	**F**	TGCACCACCAACTGCTTA
		**R**	GGATGCAGGGATGATGTTC
